# Inner Retinal Layer Hyperreflectivity Is an Early Biomarker for Acute Central Retinal Artery Occlusion

**DOI:** 10.3389/fmed.2022.854288

**Published:** 2022-07-06

**Authors:** Daniel A. Wenzel, Sven Poli, Maria Casagrande, Vasyl Druchkiv, Martin S. Spitzer, Karl Ulrich Bartz-Schmidt, Carsten Grohmann, Maximilian Schultheiss

**Affiliations:** ^1^University Eye Hospital, Centre for Ophthalmology, University Hospital Tübingen, Tübingen, Germany; ^2^Department of Ophthalmology, University Medical Center Hamburg-Eppendorf, Hamburg, Germany; ^3^Department of Neurology and Stroke, University Hospital Tübingen, Tübingen, Germany; ^4^Hertie Institute for Clinical Brain Research, University Hospital Tübingen, Tübingen, Germany

**Keywords:** central retinal artery occlusion (CRAO), optical coherence tomography, retinal ischemia, retinal imaging biomarkers, ischemia biomarker

## Abstract

**Purpose:**

To investigate inner retinal hyperreflectivity on optical coherence tomography (OCT) as a potential biomarker indicating acute central retinal artery occlusion (CRAO).

**Methods:**

A total of 56 patients at two university hospitals with acute CRAO (symptom onset ≤48 h) were included in this retrospective study. The optical intensity of the inner retinal layers was determined in both eyes and the relationship between symptom onset and inner retinal layer optical intensity in OCT scans compared to the unaffected fellow eye was analyzed. Several differential diagnoses [central retinal vein occlusion, anterior ischemic optic neuropathy, diabetic macular edema, and subretinal fibrosis/disciform scar (Junius-Kuhnt)] served as controls to validate optical intensity-based diagnosis of CRAO.

**Results:**

CRAO strongly correlated with an increased inner retinal layer hyperreflectivity in this cohort with acute CRAO with a time since symptom onset ranging from 1.1 to 48.0 h. Receiver operating characteristic (ROC) analysis showed an area under the curve of 0.99 to confirm CRAO with a true positive rate of 0.93 and a false positive rate of 0.02. No correlation between optical intensity and time since symptom onset was noticeable. None of the differential diagnoses did show an elevated optical intensity of the inner retinal layers as it was detectable in CRAO.

**Conclusion:**

OCT-based determination of inner retinal layer hyperreflectivity is a very promising biomarker for a prompt diagnosis of CRAO in an emergency setting. This may be of major interest to speed up the administration of a possible thrombolytic treatment.

## Introduction

Acute central retinal artery occlusion (CRAO) causes sudden monocular vision loss. Early intravenous thrombolysis (IVT) within 4.5 h is currently subject of ongoing prospective randomized trials. However, established algorithms and strategies for a rapid and accurate diagnosis and to triage patients do not yet exist. Most patients do not reach appropriate medical facilities in time to potentially initiate IVT, which illustrates the urgent need for retinal ischemia biomarkers in order to quickly and safely diagnose CRAO and provide therapy within a therapeutic time window of 4.5 h (1–7).

Typically, CRAO is a clinical diagnosis, though funduscopic changes, such as a cherry red spot or retinal pallor, may be lacking within the IVT-relevant very early phase, while ischemic signs may already become apparent on optical coherence tomography (OCT) scans (8). OCT provides high resolution non-invasive microstructural retinal images and has been shown to be a valuable diagnostic instrument for CRAO (9–16). Several retinal ischemia biomarkers can be visualized early, using OCT imaging within the acute phase of CRAO. Besides a loss of structure of the retinal layers, ischemic intracellular edema causes a time-dependent increase in retinal thickness, which eventually resolves and is followed by severe inner retinal atrophy in the chronic phase (9, 10, 12, 14–18). The relative retinal thickness increase (RRTI, retinal thickness increase at the thickest portion of the papillomacular bundle of the affected compared to unaffected eye) may provide information about the onset of ischemia with high accuracy (16). The ischemic edema is accompanied by hyperreflective inner retinal layers (ganglion cell layer to outer plexiform layer) and hyporeflective outer retinal layers (11, 19). Whereas the hyperreflectivity of the inner retinal layers is discussed to be most likely due to an increased intracellular ischemic edema, whereas consequently hyporeflectivity of the outer layers is caused by the decreased signal permeability of the edematous inner retinal layers (19, 20). However, as the retinal thickness often is normal or only mildly increased within the first hours (8, 10, 16), we hypothesized that the ischemia-induced increase in optical intensity may potentially be visible as the first ischemic sign anteceding retinal edema. This study therefore investigated the optical intensity of the inner retinal layers as a diagnostic criterium of CRAO and as a discriminator between potential differential diagnoses of CRAO with (sub-)acute vision loss.

## Materials and Methods

### Study Design and Patient Selection

The retrospective analysis included 56 patients (35 male, 21 female; age 73.1 ± 10.9, range 42–93) with acute CRAO who presented at two tertiary care facilities (University Eye Hospital Tübingen, Germany and Department of Ophthalmology, University Medical Center Hamburg-Eppendorf, Germany). All included patients were able to reliably report the time of symptom onset (≤48 h) of sudden, painless and persistent monocular vision loss and received an OCT scan of both eyes within 48 h [mean (SD) time-to-OCT (TTO): 13.2 ± 10.5 h (range 1.1–48.0 h)]. Visual acuity was ≤20/400. CRAO was diagnosed by an ophthalmologist. Inner retinal layer reflectivity of the affected and unaffected eye was compared. Patients with high picture noise or poor OCT image quality had to be excluded prior to analysis. Mean OCT quality index (signal-to-noise ratio, higher index indicates better quality) was 26.4 ± 5.1 in CRAO eyes and 27.4 ± 4.7 in healthy fellow eyes. Also lens status was analyzed, as potentially opacities can decrease image quality: lens status was symmetric in 55 patients. Lens status was according to age (no cataract) in 14 patients, 15 patients had incipient cataract, 7 patients presented with advanced cataract and pseudophakia was recorded in 15 patients. In four patients lens status was not recorded. One patient was pseudophakic in the healthy eye and had incipient cataract on the opposite eye affected by CRAO. Patients with retinal/macular pathologies other than CRAO (reperfused/transient CRAO, arteritic CRAO, cilioretinal artery, age-related macular degeneration, epiretinal gliosis, etc.) were also excluded.

In addition, the optical intensity of the inner retinal layers was analyzed and compared to the fellow eye in 40 patients with manifest potential differential diagnoses of possibly causing subjective acute vision loss, such as central retinal vein occlusion (CRVO; *n* = 10), diabetic macular edema (DME; *n* = 10), subretinal fibrosis/disciform scar (Junius-Kuhnt, JK; *n* = 10), or non-arteritic anterior ischemic optic neuropathy (NA-AION; *n* = 10). The inner retinal optical intensity of the eyes with CRAO was compared to the more severely affected fellow eyes of the other differential diagnoses.

### Data Collection and Statistics

**FIGURE 1:**
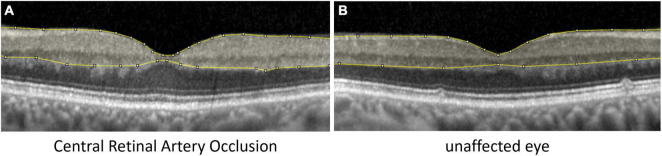
Analysis of inner retinal layer reflectivity. Segmentation of the inner retinal layers, where the optical intensity was analyzed in eyes with **(A)** central retinal artery occlusion and **(B)** the unaffected fellow eye.

Optical coherence tomography scans were performed with a Spectral-Domain OCT (Spectralis OCT, Heidelberg Engineering, Germany). Horizontal scans and the “white-on-black” mode through the fovea of both eyes were processed, saved as grayscale JPEG images and then further analyzed with ImageJ (National Institutes of Health, Bethesda, MD, United States) similar to the study of Chen et al. (11). The fovea centralis was identified and the four scans around the central scan (two below and two above) and the central scan were used for segmentation. After manual segmentation of the inner retinal layers (see Figure 1; retinal nerve fiber layer, ganglion cell layer, inner plexiform layer, inner nuclear layer, and outer plexiform layer) the raw scores of optical intensity that were inside the segmentation [gray scale, 0 (black) to 255 (white)] were extracted and analyzed using the software R (21). Only the inner retinal layer reflectivity was included into our analysis. Receiver operating characteristic (ROC) analysis was performed to classify between the eye affected and unaffected eye given the optical intensity. For each value of optical intensity sensitivity and specificity was calculated. The optimal cut-off was found by maximizing the sum of sensitivity and specificity.

Analysis of variance (ANOVA) and *post hoc t*-test adjusted for multiple comparisons with Bonferroni method were used to compare the optical intensity between the potential differential diagnoses of CRAO and test for significance. The residuals from the linear model were verified for normal distribution using Shapiro–Wilk test (*p* > 0.05). Variances were equal (Levene test *p* > 0.05).

## Results

**FIGURE 2:**
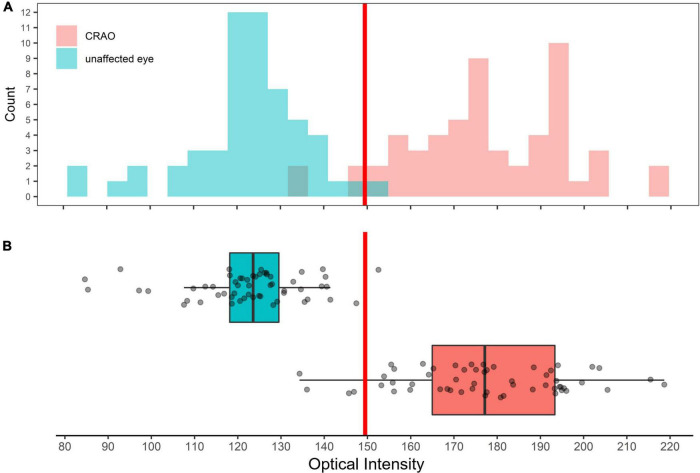
Distribution of optical intensity of in eye with central retinal artery occlusion and unaffected fellow eyes. Histogram **(A)** and box plot **(B)** showing the distribution of optical intensity in the eyes with CRAO (red) and in the unaffected fellow eyes (cyan). Differences in optical intensity were highly significant (*p* < 0.001).

**FIGURE 3:**
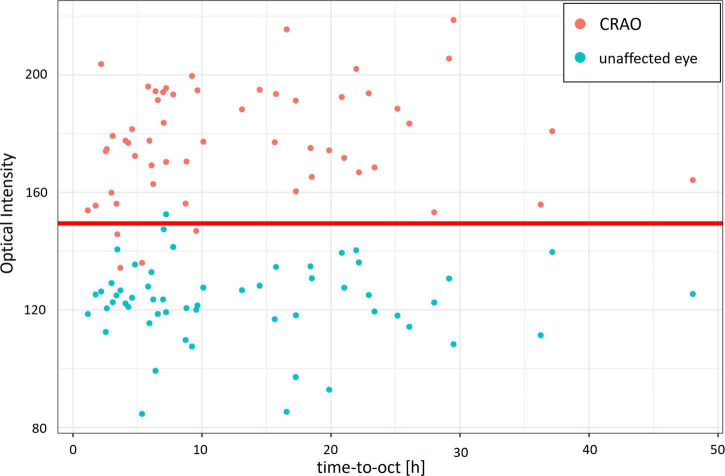
Optical intensity over time of the affected and unaffected fellow eye. Optical intensity of the inner retinal layers differs significantly in acute central retinal artery occlusion between the affected (red) and the unaffected fellow eye (cyan). Time did not have an impact or correlation on the optical intensity increase of the inner retinal layers (*R*^2^_adj_ = 0.009). The red line marks the optimal cut-off value estimated with the ROC analysis.

**FIGURE 4:**
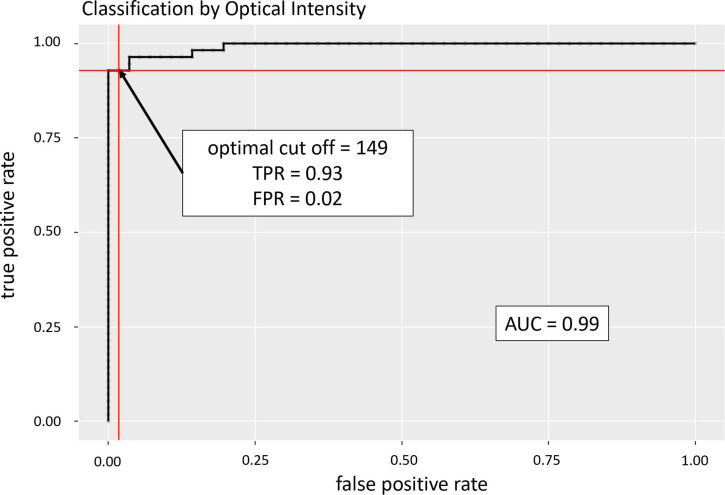
Receiver operating characteristic (ROC) analysis. ROC analysis revealed an area under the curve of 0.99 with the optimal cut-off (sum of highest sensitivity and specificity) to confirm CRAO at an optical intensity of 149.46 revealing a true positive rate (TPR) of 0.93 and a false positive rate (FPR) of 0.02.

In the analyzed patient cohort of 56 patients the time between known symptom onset and OCT scan (TTO) ranged from 1.1 to 48.0 h (13.2 ± 10.5 h (mean ± SD; see Table 1 for data overview). The optical intensity of the study eye group affected by CRAO ranged from 134 to 219 (mean 177 ± 19) compared to 85 to 153 (123 ± 14) in the group of healthy fellow eyes (see Figure 2), and, without exception, was higher in all of the patients’ eyes with CRAO compared to their fellow eye (see Figure 3). The optical intensity was normally distributed and showed a statistically highly significant difference (*p* < 0.001) between both groups. A temporal correlation of the increase in optical intensity could not be observed (*R*^2^_adj_ = 0.009), as a distinct difference in optical intensity could be seen from the very beginning (within the first hours) (see Figure 3). The mean relative increase in optical intensity of the affected compared to the fellow eye was 46.8 ± 25.5% (95% confidence interval 39.4–52.6%). ROC-analysis (see Figure 4) of the classification by inner retinal layer optical intensity revealed the optimal cut-off with the highest sum of sensitivity and specificity at a raw score of 149.46 with an area under the curve (AUC) of 0.99 and a true positive rate (sensitivity) of 0.93 and false positive rate (1-specificity) of 0.02. Using the optimal cut-off, only four CRAO retinas showed an optical intensity, which was located in the range of the healthy eyes and vice versa even only one healthy retina above 149.46 respectively in the range of CRAO retinas.

**TABLE 1:** Data overview.

	Range	Mean (SD)	Median (Q1, Q3)
Time-to-oct (h)	1.1–48.0	13.2 (10.5)	9.0 (5.2, 20.1)
Mean optical intensity (CRAO)	134–219	177 (19)	177 (165, 193)
Mean optical intensity (fellow eye)	85–153	123 (14)	124 (118, 130)
Absolute difference	5.1–130.1	54.8 (23.6)	53.6 (38.3, 68.8)
Relative increase (%)	3.7–152.5	46.8 (25.5)	43.9 (29.7, 57.1)

*Time-to-OCT in hours (time from symptom onset to the time of OCT scan), mean optical intensity in the eyes with central retinal artery occlusion and the unaffected fellow eye and the absolute and relative difference between both groups.*

**FIGURE 5:**
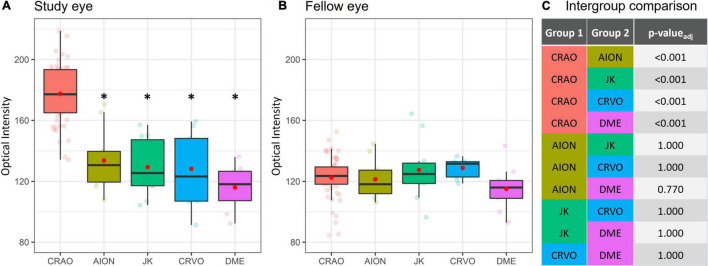
Optical intensity of potential differential diagnoses of CRAO. Optical intensity of the study eye vs. fellow eye. Eyes with CRAO showed a significant higher optical intensity compared the other analyzed groups **(A,C)**, whereas there are no differences between the groups of the fellow eyes **(B)**. In none of the investigated differentials (CRVO, central retinal vein occlusion; N-AION, non-arteritic anterior ischemic optic neuropathy; DME, diabetic macular edema; JK, Junius-Kuhnt degeneration, subretinal fibrosis/disciform scar) the inner retinal optical intensity compared to the fellow eye was significantly different **(A,B)**. *level of statistically significant difference (*p*_*adj*_ ≤ 0.05) compared to CRAO.

The comparison of optical intensity between possible differential diagnoses of CRAO showed a significant difference (see Table 2; *p* < 0.001 in all pairwise comparisons with CRAO) between CRAO and AION, JK, CRVO, and DME, whereas there were no significant differences between these groups (see Table 2; AION vs. JK, *p*_adj_ = 1.000; AION vs. CRVO, *p*_adj_ = 1.00; AION vs. DME, *p*_adj_ = 0.770, JK vs. CRVO, *p*_adj_ = 1.000; JK vs. DME, *p*_adj_ = 1.000; and CRVO vs. DME, *p*_adj_ = 1.000) or between the fellow eyes (*p*_adj_ > 0.05) (see Figure 5).

**TABLE 2:** Optical intensity data of all included groups.

	CRAO	AION	JK	CRVO	DME	*p*-Value
**Study eye**						<0.001^a^
*n*	56	9	10	9	9	
Range	134–219	108–171	104–157	91–159	79–136	
Mean (SD)	177 (19)	134 (22)	129 (19)	128 (25)	112 (19)	
Median (Q1, Q3)	177 (165, 193)	131 (120, 140)	125 (117, 147)	123 (107, 148)	112 (99, 126)	
**Fellow eye**						0.248^a^
*n*	56	9	10	9	9	
Range	85–153	106–145	96–164	119–137	93–143	
Mean (SD)	123 (14)	121 (13)	128 (20)	129 (6)	115 (15)	
Median (Q1, Q3)	124 (118, 130)	118 (112, 127)	125 (119, 132)	132 (123, 133)	116 (109, 121)	

*SD, standard deviation; Q1, first quartile that corresponds to 25%; Q3, third quartile that corresponds to 75%.*

*^a^ANOVA.*

## Discussion

Optical coherence tomography is a non-invasive high resolution *in vivo* retinal imaging method and is likely to become the key diagnostic instrument in the acute management of CRAO. Typically, CRAO is a clinical diagnosis, but as unfortunately clinical signs such as retinal edema and a cherry red spot may have not evolved in the very early phase, pathognomonic signs on OCT scans can provide valuable information about CRAO. An increase in retinal thickness for example is a characteristic feature, for which a temporal correlation has been shown recently (16, 17). Similarly, a rise in optical intensity in the affected eye can be observed (9, 11, 12, 19), but the temporal dynamics have yet been unknown. In CRAO the inner retinal layers appear as a hyperreflective demarcated and mostly edematous band. Consequently, the light signal is attenuated by edematous inner retinal layers and the outer retinal layers appear hyporeflective. Possibly, the optical intensity is correlated to the final visual outcome (11).

In this study we retrospectively analyzed the optical intensity of the inner retinal layers (analyzed collectively) compared to the healthy fellow eye in patients that had suffered from CRAO. The optical intensity in the CRAO group was statistically highly significant higher compared to the healthy fellow eye group (*p* < 0.001; AUC 0.99, true positive rate 0.93, and false positive rate 0.02), which can therefore be interpreted as reliable parameter to diagnose CRAO in the acute phase. The absolute values in our study of the measured optical intensity of the inner retinal layers are comparable to the data of previously published studies (11, 12). Another study reported that the increase in optical intensity of the inner nuclear layer was most indicative for CRAO among all retinal layers (19). However, in our opinion defining single layers of the inner retina can be quite challenging in the acute situation as they become hard to distinguish in the ischemic retina. We therefore preferred the summarized analysis of the optical intensity of the inner retina as a whole.

The first 4.5 h after ischemia onset are critical for the prognosis of every therapeutic approach. Administration of IVT within 4.5 h shows a superior visual prognosis compared to the untreated natural course (4, 6, 7). The RRTI, the change of retinal thickness in the affected compared to the healthy fellow eye, is a parameter showing temporal changes and therefore allowing a highly accurate estimate of ischemia duration, which can be essential when the exact onset of symptoms cannot be reported reliably (10, 16, 17). Furthermore, the RRTI might reveal the amount ischemia induced retinal damage (higher RRTI represents more severe damage). Nevertheless, since significant retinal edema develops over time and may be lacking in the very early phase of CRAO, the RRTI might not be suitable to diagnose CRAO within the first few hours (16, 17). Moreover, retinal edema is not a specific sign of ischemia and can be apparent in other retinal vascular disorders such as DME or retinal vein occlusion. Vice versa, this is where the optical intensity comes in to the picture as the optical intensity allows quick diagnosis of CRAO and may therefore enhance the diagnostic algorithm as an early biomarker – even in the absence or before the development of significant retinal edema. In contrast to the time-dependent RRTI, the optical intensity did not show a significant temporal increase or correlation (16, 17). The increase in optical intensity was already visible in patients with a very short time since symptom onset (1–4 h) and hyperreflectivity did not change significantly over time (up to 48 h). Consequently, optical intensity comes with the great potential to be used as an early diagnostic discriminator in patients with acute vision loss in order to decide whether the underlying cause is CRAO, and thus requires an immediate neurovascular workup. Potential differential diagnoses coming with a possible monocular loss or reduction of vision, such as CRVO, DME, JK, or NA-AION can be ruled out safely. Vitreous hemorrhage, another potential differential, can be easily confirmed by fundoscopy or sonography, as OCT typically is not possible due to limited light signal penetration.

Among the limitations of this study is the retrospective character including a possible selection bias, as only patients with known symptom onset and available OCT scans of both eyes were included in our analysis. The central scan through the fovea as well the two scans above and below were used for segmentation, which could be a possible bias but enables an automatized workflow for the future detection of CRAO, because the central scan is usually detected automatically by OCT imaging machines. Also, the relatively low number of patients within the first 3 h limit the validity of the optical intensity increase within the acute phase, as it remains unknown when exactly and how fast the optical intensity increases, but nonetheless there was no significant increase with time in our cohort. Moreover, quality parameters of OCT imaging need to be established for a valid optical intensity analysis as image noise may reduce the reliability of optical intensity. For example, a lower limit of the signal-noise-ratio to warrant valid use of optical intensity needs to be established. Opacities of the optical axis such as cataract or corneal opacities, vitreous hemorrhage can decrease the light signal and therefore it needs to be kept in mind that this may potentially influence the measured optical intensity. This is especially relevant in patients with asymmetrical lens status. Although we could differentiate CRAO from healthy eyes accurately in almost all, there are a few patients that have not matched perfectly: one patient with asymmetrical lens status was included in our study: the eye with CRAO had incipient cataract, the healthy fellow eye was pseudophakic and showed increased optical intensity compared to the other healthy eyes. However, there we found no particular reason why this was not the case in other pseudophakic eyes. Other eyes with an optical intensity out of line were found in three CRAO eyes, in which the optical intensity was too low compared to the other eyes with CRAO: one patient had a relatively low RRTI of only 7% 3.5 h after ischemia onset and therefore could somewhat influence the optical intensity. The two other patients (one with both eye having incipient cataract, one with both eyes having advanced cataract) had a high image noise as a possible confounder, although signal-to-noise ratio reached a decent quality level in these patients. OCT enables a fast visualization of characteristic microstructural changes in the ischemic retina in CRAO (9, 14, 20). This study revealed that the diagnosis of CRAO on the basis of an optical intensity increase in the inner retinal layers is reliable, even in the acute phase where other clinical signs may only be visible faintly. Further research with a higher number of patients is required to confirm this studies results and the clinical use in order to implement optical intensity-based diagnosis of CRAO in an emergency algorithm. Included in an emergency algorithm, the optical intensity should be used to decide on whether the diagnosis is CRAO or not and the RRTI to define or confirm the time since ischemia onset. Altogether, both parameters support patient-reported information with objectifiable information, that potentially could be decisive in the further management and visual prognosis. Moreover, an automatic determination of these parameters by an automated OCT software or machine learning algorithms would greatly accelerate the ophthalmological diagnosis and further neurovascular referral to promptly initiate IVT within 4.5 h of ischemia onset and/or to start with a comprehensive neurovascular work-up. Additional imaging modalities could potentially enhance the diagnostic workflow. There is evidence that a positive retrobulbar spot sign visualized by ultrasound could be associated with a poor response to IVT and could therefore be a contraindication (15, 22, 23). Retinal diffusion restriction visualized by diffusion-weighted magnetic resonance imaging (DWI-MRI) may provide additional information (24). Further studies are needed to explore the possibilities offered by different diagnostic tools. Conclusively, a hyperreflectivity of the inner retinal layers may confirm acute CRAO. Particularly, when determined in the very early phase, the optical intensity may serve as a diagnostic biomarker, also in the absence of obvious fundus changes or before retinal edema can be detected.

## Data Availability Statement

The raw data supporting the conclusions of this article will be made available by the authors, without undue reservation.

## Ethics Statement

Ethical review and approval was not required for the study on human participants in accordance with the local legislation and institutional requirements. Written informed consent for participation was not required for this study in accordance with the national legislation and the institutional requirements.

## Author Contributions

DW, MSS, KB-S, CG, and MS contributed to the conception and design of the study. DW, MC, CG, and MS performed the data collection. VD and CG performed the statistical analysis. DW, SP, MSS, CG, and MS interpreted the data. DW and MS searched literature. DW wrote the first draft of the manuscript. VD, CG, and MS wrote sections of the manuscript. DW, SP, MC, VD, MSS, KB-S, CG, and MS performed the manuscript revision and approved for submission and publication. All authors contributed to the article and approved the submitted version.

## Conflict of Interest

MS, MSS, and SP are initiators of the REVISION trial on early intravenous thrombolysis in central retinal artery occlusion (NCT04965038), study medication (verum and placebo) is supplied by Boehringer Ingelheim Pharma GmbH & Co. KG. The remaining authors declare that the research was conducted in the absence of any commercial or financial relationships that could be construed as a potential conflict of interest.

## Publisher’s Note

All claims expressed in this article are solely those of the authors and do not necessarily represent those of their affiliated organizations, or those of the publisher, the editors and the reviewers. Any product that may be evaluated in this article, or claim that may be made by its manufacturer, is not guaranteed or endorsed by the publisher.

## Abbreviations

CRAO: central retinal artery occlusion
CRVO: central retinal vein occlusion
DME: diabetic macular edema
JK: Junius-Kuhnt, age related macular degeneration with subretinal fibrosis/disciform scar
(NA-)AION: (non-arteritic) anterior ischemic optic neuropathy
OCT: optical coherence tomography
RRTI: relative retinal thickness increase.
